# The regulatory role and therapeutic potential of long non-coding RNA in non-small cell lung cancer

**DOI:** 10.7150/jca.103182

**Published:** 2025-01-06

**Authors:** Sunming Xia, Xuean Lu, Weier Wang, Xinyi Pan, Jiaqi Cui, Shengjie Wang, Zhao Wang

**Affiliations:** 1 Donghai County People's Hospital affiliated to Kangda College of Nanjing Medical University, Lianyungang 222300, Jiangsu, China.; 2 Department of General Surgery, Donghai County People's Hospital, Lianyungang 222300, Jiangsu, China.; 3 School of Medicine, Nanjing University of Chinese Medicine, Nanjing 210023, Jiangsu, China.; 4 Department of Basic Medicine, Kangda College of Nanjing Medical University, Lianyungang 222000, Jiangsu, China.; 5 Department of Oncology, The First Affiliated Hospital of Soochow University, Suzhou 215006, Jiangsu, China.

**Keywords:** LncRNAs, proliferation, metastasis, targeted therapies, NSCLC

## Abstract

Lung cancer remains the leading cause of cancer-related mortality worldwide, with non-small cell lung cancer (NSCLC) being the predominant subtype. Recent advances in transcriptome sequencing have highlighted the critical role of long non-coding RNAs (lncRNAs) in NSCLC, with lncRNAs influencing gene expression through epigenetic, transcriptional, and post-transcriptional mechanisms. Despite the growing understanding of lncRNAs, challenges such as delayed diagnosis and drug resistance continue to complicate NSCLC management. This review explores novel findings in the role of lncRNAs (e.g., MALAT1, HOTAIR, and GAS5) in NSCLC, with a particular focus on their encoded small peptides and N6-methyladenosine (m6A) modifications. We further discuss how the interplay between lncRNAs, their encoded peptides, and m6A modifications can provide new strategies for improving NSCLC diagnosis, treatment, and overcoming drug resistance. This review also highlights emerging research avenues that could lead to innovative clinical interventions in NSCLC.

## Introduction

Lung cancer remains the leading cause of cancer-related mortality worldwide, accounting for approximately one in five cancer deaths^1^, ^2^. Non-small cell lung cancer (NSCLC) constitutes approximately 80-85% of all lung cancer cases^3^. The poor survival rates of patients with lung cancer are primarily due to the lack of effective early diagnostic biomarkers and the development of resistance to chemotherapy or targeted therapies in the advanced stages of the disease^4^, ^5^. Recent advancements in molecular biology have highlighted the critical role of non-coding RNAs (ncRNAs) in gene expression regulation and their implications in cancer biology, including lung cancer. NcRNAs are categorized based on nucleotide length into long non-coding RNAs (lncRNAs) and small or short non-coding RNAs. LncRNA, typically exceeding 200 nucleotides in length, represent one of the largest and most diverse families of RNAs. Despite their lack of protein-coding ability, lncRNAs make up a substantial portion of the non-coding RNA population^6^-^8^. Recent studies have demonstrated that the dysregulation, deletion or mutation of lncRNAs is closely associated with various diseases, including lung cancer. LncRNAs regulate gene expression through multiple mechanisms, including epigenetic modifications^9^, mRNA splicing^10^, interactions with microRNA^11^, lncRNA-protein interactions^12^, and lncRNA-mRNA interactions^13^. Furthermore, the regulatory mechanisms of lncRNA in tumor cell progression vary depending on their intracellular localization. Nuclear lncRNAs can influence biological functions by modulating chromatin structure and transcription and serving as structural scaffolds to anchor nuclear domains, thereby regulating biological processes^14^. Contrastingly, cytoplasmic lncRNAs often impact cell signaling by regulating post-transcriptional modifications and mRNA translation^15^. Currently, accumulating studies have revealed that certain lncRNAs can encode small open reading frame (sORF)-derived peptides, which are believed to contribute to the sensitivity of cancer treatment^16^. Additionally, RNA modification at the N6 position of internal adenosine (m6A) in ncRNAs by the methyltransferase is essential for malignant maintenance of tumor cells^17^. In summary, the regulatory roles of lncRNAs in NSCLC are multifaceted and profound. Understanding these roles not only provides insight into the molecular underpinnings of NSCLC but also opens up new avenues for diagnostic and therapeutic innovations. This review explores the diverse functions of lncRNAs in NSCLC, emphasizing their mechanisms of action, impact on tumor biology and potential as therapeutic targets.

## NSCLC-linked lncRNAs

In lung cancer, lncRNAs play pivotal roles in both oncogenic and tumor-suppressive pathways. They regulate key processes such as cell proliferation^18^, apoptosis^19^, metastasis^20^, and angiogenesis^21^, contributing to the complexity of the tumor microenvironment^22^, ^23^. Dysregulated expression of specific lncRNAs, including MRPL23-AS1, MALAT1 and GAS5, has been associated with tumor progression, poor prognosis and therapy resistance. For instance, MALAT1 promotes cell migration and invasion^24^, while MRPL23-AS1 is involved in EMT and metastasis^25^. Conversely, GAS5 functions as a tumor suppressor by inducing apoptosis and inhibiting cell growth^26^. The regulatory mechanisms of lncRNAs are diverse and involve interactions with DNA, RNA and proteins to modulate key signaling pathways. LncRNAs may act as molecular scaffolds, decoys, guides, or enhancers, influencing gene expression at epigenetic, transcriptional and post-transcriptional levels^27^-^29^. This multifaceted regulatory capability highlights the importance of lncRNAs as central nodes within the intricate networks that drive lung cancer development and progression.

## LncRNAs and the hallmarks of NSCLC

LncRNAs are integral to several hallmarks of NSCLC, including cell proliferation, metastasis, apoptosis and angiogenesis. Their regulatory roles in these processes are crucial for understanding lung cancer progression and developing targeted therapies. The regulation of lncRNA expression and function in cancer often mirrors the mechanisms that govern known oncogenes and tumor suppressors, including DNA methylation, gene amplification or deletion or mutations. Furthermore, advances in bioinformatics and biochemical methodologies have revealed that ncRNAs previously considered noncoding may actually encode small biologically active peptides. These coding products play a significant role in the progression of NSCLC. This section will highlight examples of well characterized lncRNAs that exhibit oncogenic or tumor-suppressive roles in NSCLC.

### Cell proliferation and cell cycle regulation

**Figure 1:**
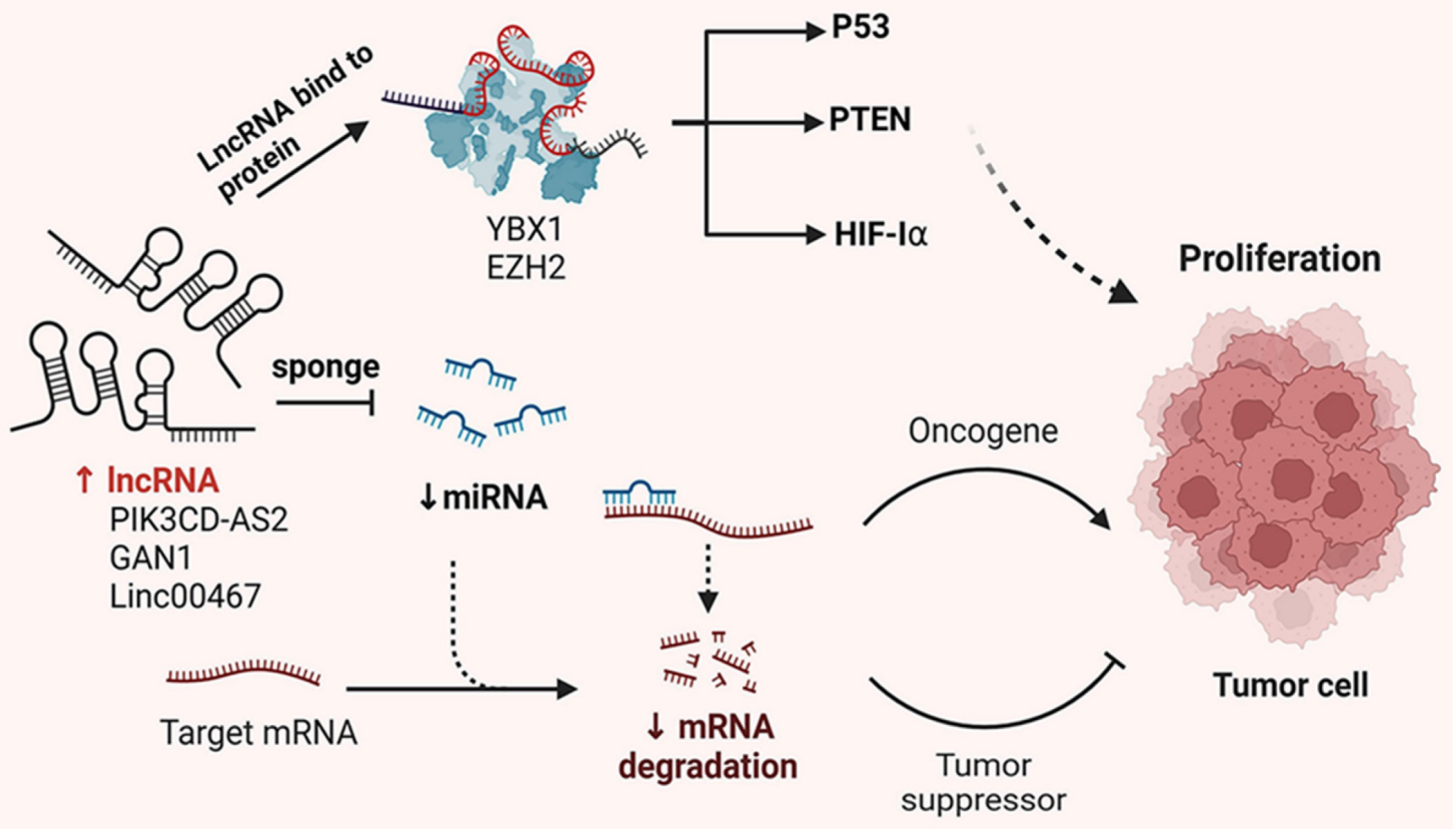
Mechanisms of lncRNA regulation of NSCLC proliferation. In NSCLC cells, lncRNAs carry specific sequences that can adsorb miRNAs, which act like sponges, thus preventing miRNAs from binding to their targets. Conversely, lncRNAs interact with proteins, affecting their post-translational modifications, protein stability, transcriptional and translational activities, ultimately affecting the proliferation of tumor cells.

The development and progression of malignant tumors are often marked by dysregulation of the cell cycle and apoptotic signaling pathways. Emerging evidence suggests that lncRNAs play either promotive or inhibitory roles in tumor development and progression. Like protein-coding genes, lncRNAs can be categorized into oncogenic and tumor-suppressive subtypes. This section provides an overview of the molecular mechanisms by which lncRNAs influence tumor proliferation, including their functions as miRNA sponges, protein scaffolds and coding peptides (Figure 1).

LncRNA-XIST is aberrantly expressed in various tumor and promotes malignant biological behavior. Zhou *et al.* demonstrated that XIST competes with miR-16 through the ceRNA mechanism, thereby reversing its suppression of the downstream target gene CDK8. This interaction leads to cell cycle arrest of NSCLC cells in the G0-G1 phase, ultimately promoting proliferation and tumor progression^30^. Additionally, RIP experiments have shown that XIST and miR-186-5p are enriched in Ago2 immuno-precipitation. XIST binds to miR-186-5p, inhibiting its tumor-suppressive effects and affecting cell proliferation through cell cycle regulation^31^. Chen *et al.* reported that lncRNA-HOTAIR is upregulated in NSCLC cell lines. Silencing HOTAIR significantly inhibits A549 proliferation by promoting miR-217 expression^32^. Furthermore, Li *et al.* found that HOTAIR acts as a competitive endogenous RNA for miR-149-5p, thereby promoting the growth and invasion of NSCLC cells^33^. MALAT1, one of the earliest identified dysregulated lncRNAs in NSCLC, is highly expressed in most tumor tissues. MALAT1 enhances cell proliferation by modulating the expression of cell cycle-related genes. It interacts with various transcription and splicing factors to promote the transition from the G1 to S phase, facilitating rapid cell division. Li *et al.* observed that MALAT1 was significantly overexpressed in various NSCLC cell lines. The knockdown of MALAT1 in A549 and H460 cell lines significantly increased miR-124 levels, which also inhibited MA-LAT1 expression. Experimental results showed that MALAT1 and miR-124 were highly enriched in Ago2, indicating their interaction. Therefore, the MA-LAT1/miR-124/STAT3 pathway promotes NSCLC cell proliferation^34^. Another study found that both MALAT1 and MDM4 were highly expressed in A549 and H460 cell lines, with a positive correlation in NSCLC tissues. MALAT1 enhances the expression of its target gene MDM4 by downregulating miR-185-5p levels, thereby promoting NSCLC cell proliferation^35^. DANCR is significantly overexpressed in NSCLC and enhances the binding of EZH2 to p21, increasing H3K27me3 modification at the p21 promoter region, which inhibits p21 expression and promotes NSCLC cell proliferation^36^. Yu *et al.* reported that DANCR competes with miR-216a to regulate β-catenin expression, thus promoting cell proliferation via the Wnt/β-Catenin signaling pathway^37^. Meng *et al.* found that JPX interacts with miR-145-5p and upregulates downstream cyclin D2 expression through the ceRNA mechanism, thus regulating cell cycle progression^38^. Additionally, SNHG15 binds competitively to miR-486, leading to upregulation of CDK14 mRNA expression and affecting NSCLC cell growth and proliferation^39^. XIST has been shown to upregulate the anti-apoptotic factor Bcl-2 by binding to miR-449a, thereby inhibiting cell apoptosis^40^. Conversely, Wu *et al.* reported that MEG3 binds to miR-7-5p, inhibits Bcl-2 expression and promotes BAX expression, thereby inducing apoptosis in NSCLC cells^41^. Overall, these studies indicate that lncRNAs play a crucial role in regulating NSCLC cell proliferation, cell cycle and apoptosis. A summary of these mechanisms is presented in Table 1.

### Metastasis and invasion

**Figure 2:**
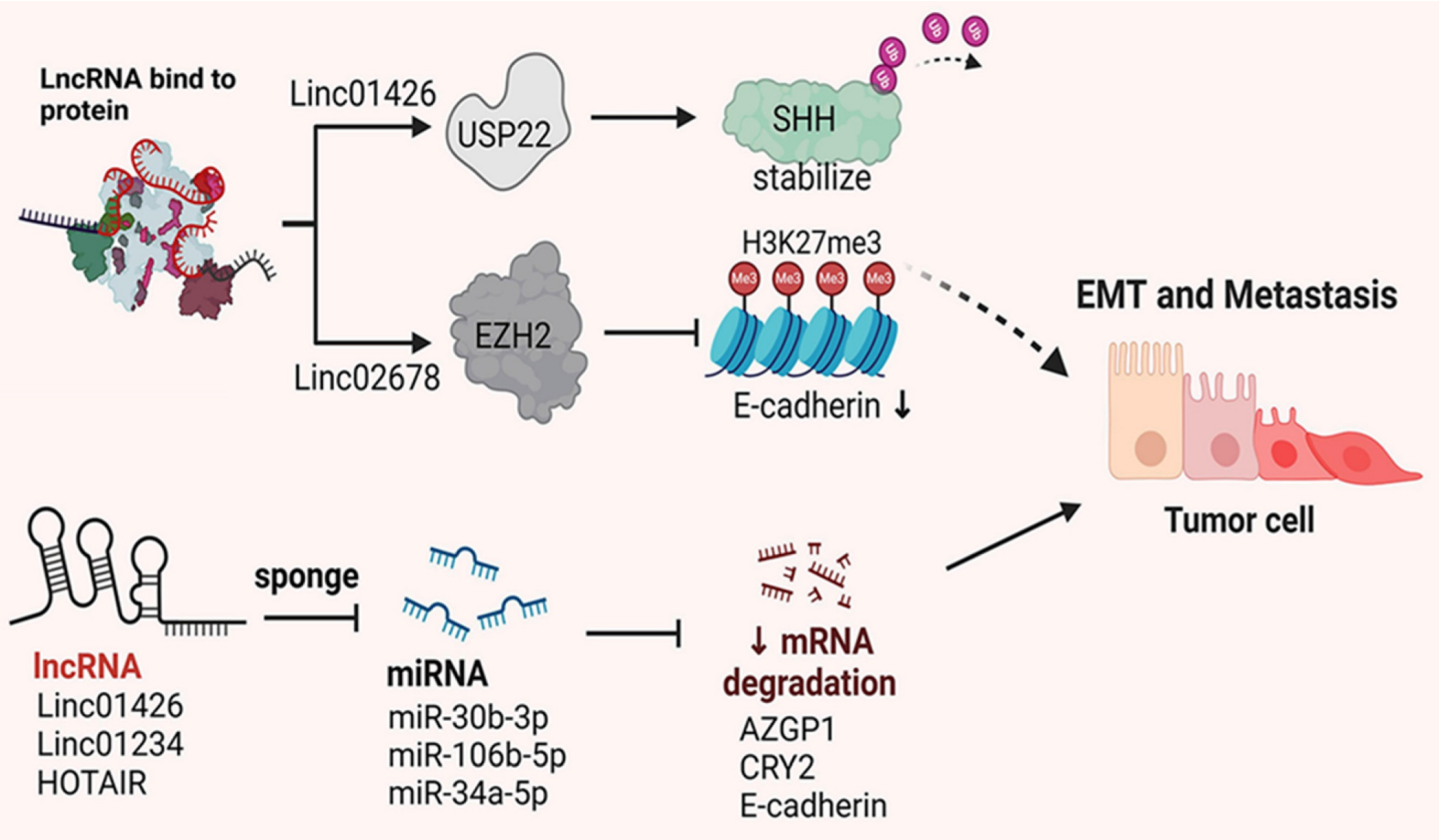
The abnormal expression and interaction of lncRNAs influence NSCLC metastasis by regulating various ceRNA networks and post-translational modifications.

Metastasis and invasion of tumor cells are critical factors contributing to the poor prognosis of patients with NSCLC. Numerous studies have demonstrated that lncRNAs are involved in regulating cell migration and invasion. This section summarizes and discusses lncRNAs associated with tumor cell migration and invasion (Figure 2).

SNHG6 is significantly overexpressed in NSCLC tissues and cells. Knockdown of SNHG6 inhibits NSCLC cell invasion. SNHG6 competitively binds to miR-944 and miR-181d-5p, promoting the expression of the transcription factor ETS1. ETS1, in turn, binds to the WIPF1 promoter region, enhancing its expression and promoting NSCLC cell invasion by regulating RhoA^60^. High SNHG6 expression is also associated with advanced pathological stage and lymph node infiltration in patients with NSCLC and can serve as an independent predictor of tumor recurrence. In A549 cells, SNHG6 binds to miR-101-3p, downregulating its expression and promoting the expression of its downstream target CDYL, which enhances the invasion ability of NSCLC cells^61^.

MEG3 and GAS5 are known tumor suppressor lncRNAs 62. MEG3 is expressed at low levels in NSCLC tissues, and its upregulation inhibits NSCLC cell migration and invasion while promoting apoptosis through p53^63^. Lv *et al.* found that MEG3 inhibits migration and invasion of NSCLC cells and enhances PTEN expression via the PI3K/AKT signaling pathway, suggesting its potential as a novel therapeutic target for NSCLC^64^. GAS5, another tumor suppressor in NSCLC, exerts effects through both p53-dependent and p53-independent pathways^65^. Dong *et al.* reported that the downregulation of GAS5 significantly promotes the growth, migration and invasion of NSCLC cells, while the miR-205/PTEN axis can partially counteract these effects. GAS5 upregulation inhibits NSCLC growth, migration and invasion through the miR-205/PTEN axis^66^. Studies have shown that LNC00673 interacts with LSD1 at the NCALD promoter region, promoting H3K4me2 demethylation, which suppresses NCALD transcription and reduces protein expression. This enhances the metastatic capability of NSCLC cells^67^. LSINCT5 interacts with the metastasis-associated transcription factor HMGA2, protecting it from proteasome-mediated degradation and increasing NSCLC cell migration^68^. Furthermore, NKILA was found to be downregulated in NSCLC tissues and correlates with lymph node metastasis and TNM staging. Mechanistically, NKILA transcription is regulated by the TGF-β signaling pathway, which inhibits NF-κB activity by reducing IKBα phosphorylation, thereby suppressing NSCLC cell migration and invasion^69^. Additionally, NKILA can inhibit EMT by modulating the IL-11/STAT3 signaling pathway 70. Overall, these studies indicate that lncRNAs play significant roles in regulating NSCLC cell EMT, invasion and metastasis. A summary of these mechanisms is presented in Table 2.

### Apoptotic response and cell death

LncRNAs play crucial roles in regulating apoptosis in NSCLC. Apoptosis can be categorized into extrinsic and intrinsic pathways. In the extrinsic pathway, death receptors such as TNFR, Fas/CD95 and TRAIL interact with extracellular ligands like TNF and FasL, leading to the sequential activation of death receptor procaspase-8, caspase-8 and caspase-3. This cascade ultimately regulates DNA fragmentation factors and caspase-activated DNases, culminating in apoptosis^79^, ^80^. In the intrinsic pathway, cytotoxic stimuli, including carcinogenic stress, chemotherapy drugs and developmental signals selectively activate BH3 family members while suppressing Bcl-2 proteins. This activation triggers pro-apoptotic effectors BAX and BAK, disrupting the outer mitochondrial membrane^81^. The resulting release of cytochrome C and ATF endonuclease from the mitochondria initiates the formation of the apoptosome through the bindings of cytochrome C to APAF1, which subsequently activates caspase-9^82^. Activated caspase-9 then induces caspase-3, caspase-6 and caspase-7, leading to protein cleavage and the execution of apoptosis. Numerous lncRNAs are involved in modulating these apoptotic processes^83^. GAS5, a well-established tumor suppressor lncRNA, induces apoptosis by sequestering the glucocorticoid receptor, thereby inhibiting its anti-apoptotic effects and increasing the sensitivity of cancer cells to apoptotic signals. Conversely, HOTAIR inhibits apoptosis by upregulating anti-apoptotic genes, which contribute to tumor survival and resistance to therapy. Han *et al.* found that lnc0218 is upregulated in NSCLC and promotes cell proliferation by regulating the miR-4677-3p/SEC61G axis, thereby suppressing apoptosis. Notably, the knockdown of lnc0218 enhances apoptosis in NSCLC cells^84^. Wang *et al.* discovered that lncRNA-ATB promotes NSCLC cell apoptosis by inhibiting miR-200a expression, which leads to the upregulation of β-catenin expression^85^. Zhang *et al.* demonstrated that XIST upregulates the anti-apoptotic factor Bcl-2 by binding to miR-449a, thereby inhibiting cell apoptosis^40^. While some lncRNAs inhibit apoptosis, others promote it. For example, Wu *et al.* showed that MEG3 binds to miR-7-5p, suppressing Bcl-2 expression and promoting BAX expression, thereby inducing apoptosis in NSCLC cells^86^. Pei *et al.* found that the lncRNA AFAP1-AS1 encodes a small peptide called ATMLP, which localizes to mitochondria and promotes the malignant progression of NSCLC. ATMLP (not the lncRNA itself) is a driver of tumor progression, and the higher the level of ATMLP in a patient's serum, the worse the prognosis. Moreover, its translation process is regulated by m6A methylation. Mechanistically, ATMLP binding to NIPSNAP1 disrupts its translocation within mitochondria and affects the process of tumor cell apoptosis^87^.

## LncRNA regulates drug resistance in NSCLC cells

**Figure 3:**
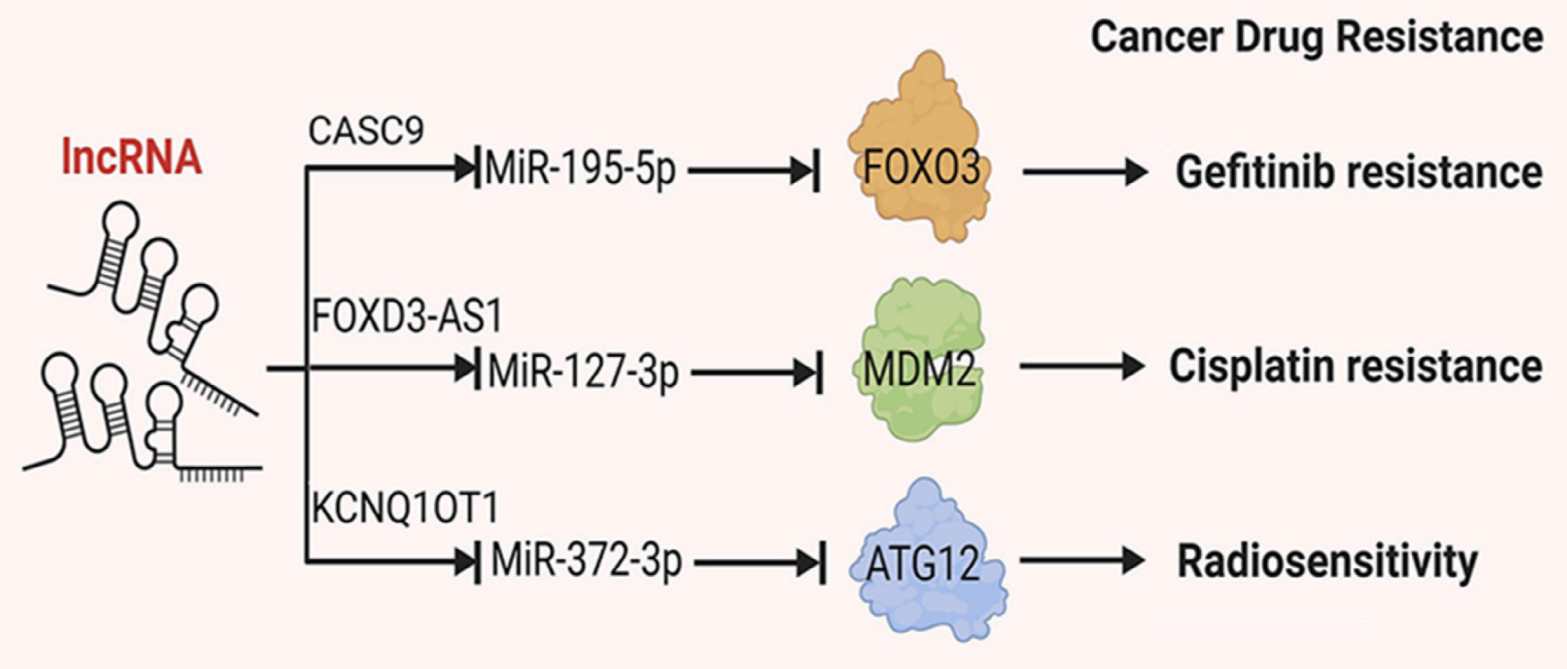
Mechanisms by which lncRNAs regulate tumor angiogenesis. Certain lncRNAs have sponge-like properties, wherein they absorb miRNAs, inhibit the binding of miRNAs to target genes and reduce degradation of target mRNAs, thereby affecting tumor drug resistance.

Due to the subtle early symptoms of NSCLC, many patients are diagnosed with metastasis at the time of initial presentation, making surgical resection unfeasible. For patients with NSCLC who are ineligible for surgery, chemotherapy and targeted therapy drugs are essential treatment modalities. However, the development of drug resistance and reduced tolerance to treatment significantly diminishes treatment efficacy and overall survival rates^88^, ^89^. LncRNAs have been implicated in the development of drug resistance in NSCLC. For instance, certain lncRNAs have been shown to influence specific drug pathways, including those targeted by tyrosine kinase inhibitors (TKIs). Notably, the lncRNA HOTAIR has been found to be upregulated in NSCLC cells that have developed resistance to TKIs. HOTAIR is thought to regulate the expression of genes involved in drug metabolism and efflux, thereby contributing to the resistance phenotype. This section summarizes and discusses the roles of lncRNAs in cellular drug resistance (Figure 3).

Platinum-based chemotherapy regimens are foundational in the clinical treatment of NSCLC, making it crucial to understand the role of lncRNAs in platinum resistance. Li *et al.* demonstrated that in Cisplatin-resistant A549 and H460 cells, the expression levels of UCA1, miR-495 RNA and NRF2 protein differed significantly from those in non-resistant cells. Mechanistically, UCA1 directly binds to miR-495, leading to its downregulation. As NRF2 is a direct target of miR-495, its expression is negatively correlated with miR-495 levels. Thus, UCA1 mediates platinum resistance in NSCLC cells through the UCA1/miR-495/NRF2 pathway^90^. Additionally, Wang and Gao showed that LINC01116 is associated with Cisplatin resistance in lung adenocarcinoma^91^. Furthermore, Yao indicated that LINC01116 influences Cisplatin resistance by regulating iron metabolism and AKT signaling in NSCLC^92^, ^93^. Additionally, lncRNAs also contribute to resistance to other chemotherapeutic agents. Pan *et al.* found that lnc-ROR is elevated in tumor tissues from docetaxel-resistant patients. *In vitro* experiments revealed that lnc-ROR affects NSCLC cell sensitivity to docetaxel through the lnc-ROR/miR-145/FSCN1 pathway^94^.

Targeted therapies are becoming increasingly important in NSCLC treatment. Resistance to these therapies is often mediated by lncRNAs. For example, over 46% of patients with NSCLC having EGFR mutations respond to EGFR-TKIs such as erlotinib and gefitinib^95^, ^96^. In EGFR-TKI resistant cells, various lncRNAs are aberrantly expressed and participate in modulating resistance to EGFR-TKIs^97^. In EGFR-TKI-resistant cells, various lncRNAs are aberrantly expressed, modulating resistance to EGFR-TKIs 94. In gefitinib-resistant NSCLC cells (e.g. SPC-A1 and H1299), researchers identified five dysregulated lncRNAs: three upregulated (UCA1, NEAT1 and CASC9) and two downregulated (EWAST1 and LNC00524). Mechanistically, the co-expression of CASC9 and EWAST1 regulates multiple pathways, including cell growth and apoptosis, thus influencing EGFR-TKI sensitivity^97^-^99^.

Chen *et al.* found that in gefitinib-resistant PC9 cells, CASC9 (candidate tumor susceptibility gene 9) binds specifically to EZH2, facilitating its binding to the DUSP1 promoter, upregulating H3K27me3 levels and suppressing DUSP1 protein expression. This process enhances NSCLC resistance to gefitinib^100^. Similarly, in extracellular vesicles released from gefitinib-resistant PC-9 cells, UCA1 is upregulated. Xu *et al.* found that in gefitinib-resistant tumor cells, UCA1 binds directly to EZH2, increasing H3K27me3 levels at the CDKN1A promoter region and reducing downstream CDKN1A expression, thereby mediating gefitinib resistance^101^. These findings highlight the importance of lncRNAs in drug resistance and support potential therapeutic targets. Further studies indicate that KCNQ1OT1 plays a significant role in radiotherapy resistance. Wang *et al.* demonstrated that KCNQ1OT1 promotes cell proliferation, migration and invasion by modulating the miR-129-5p/JAG1 pathway^102^. Additionally, KCNQ1OT1 influences cell proliferation, autophagy and apoptosis through the miR-204-5p/ATG3 pathway, contributing to radiotherapy resistance in lung adenocarcinoma^103^. Furthermore, He *et al.* found that KCNQ1OT1 is significantly upregulated in radiotherapy-resistant A549 and H1975 cells. KCNQ1OT1 binds to miR-372-3p, suppressing its expression and promoting the expression of downstream ATG5 and ATG12 mRNA and proteins, which reduced NSCLC cells' sensitivity to radiotherapy^104^.

Moreover, TTTY15 inhibits the binding of DNMT3A to the TBX4 promoter, thereby regulating TBX4 expression and affecting NSCLC cells' sensitivity to radiotherapy^105^. Jiao *et al.* discovered that lncRNA SNHG14 regulates HMGB1 expression by sponging miR-34a. Silencing SNHG14 enhances the sensitivity of NSCLC cells to carboplatin, suggesting SNHG14 is a potential target to overcome drug resistance^106^. Interestingly, lncRNAs can both induce and inhibit drug resistance in tumor cells^107^. For instance, lncRNA FENDRR binds directly to the 3' untranslated region (3'UTR) of the MDR1 gene, preventing RNA-binding proteins from associating with the MDR1 3'UTR, reducing MDR1 expression, and inhibiting drug resistance^108^. Nakano *et al.* identified LNC00460 as a novel marker associated with poor response and prognosis in EGFR-TKI therapy. In lung cancer cells, LNC00460 enhances EGFR-TKI resistance by acting as a competitive endogenous RNA (ceRNA) for miR-149-5p, promoting interleukin-6 expression and inducing an EMT-like phenotype. Knocking out LNC00460 in gefitinib-resistant NSCLC cells restore their sensitivity to EGFR-TKI. Moreover, high LNC00460 expression correlates with shorter progression-free survival (PFS) and overall survival (OS) after gefitinib treatment^109^. Wang *et al.* found that LINC01116 is upregulated in gefitinib-resistant NSCLC cells and tissues. Silencing LINC01116 increases IFI44 expression, and IFI44 overexpression reverses gefitinib resistance in PC9/R cells, indicating that LINC01116 modulates gefitinib resistance partly through IFI44^43^. Additionally, Li *et al.* reported that RHPN1-AS1 is downregulated in gefitinib-resistant patients and NSCLC cell lines, with this downregulation linked to poor prognosis. Overexpression of RHPN1-AS1 restores gefitinib sensitivity in resistant NSCLC cells by interacting directly with miR-299-3p to positively regulate TNFSF12 expression, modulating gefitinib resistance^110^. Additionally, lncRNAs such as SNHG15^111^, CASC9^112^, LNC554202^113^ and BLACAT1^114^ also significantly impact resistance to EGFR-TKI therapy. Understanding the interaction mechanisms between lncRNAs and drug resistance can provide new therapeutic strategies to enhance treatment efficacy. A summary of some of the mechanisms is presented in Table 3.

## The advantage and challenge of therapeutic targeting of lncRNA

Targeted lncRNA therapy offers a novel and promising strategy for the treatment of NSCLC. The precise modulation of lncRNAs involved in tumor progression and metastasis could offer new avenues for effective cancer therapies^124^, ^125^. Over recent decades, substantial clinical investments have been directed toward RNA-based therapeutic approaches. Presently, there are several RNA-based therapeutic modalities available, including antisense oligonucleotides (ASOs), RNA interference (RNAi), small molecule inhibitors, microRNA mimics, and CRISPR-Cas9 gene editing. Among these, ASOs and siRNAs are the most extensively utilized^126^, ^127^. Preclinical studies have shown that synthetic miRNA-based therapeutic molecules, combined with various protective coating techniques, have enabled effective delivery and anti-tumor activity^128^, ^129^. MALAT1 was the first lncRNA reported to be associated with tumor metastasis. In a mouse model of metastatic NSCLC, subcutaneous injection of MALAT1 ASO significantly reduced the burden of lung nodules, indicating the therapeutic potential of targeting MALAT1 in metastatic lung cancer^130^, ^131^. Similarly, in patients with metastatic renal cell carcinoma, high expression of the lncRNA ARSR is associated with shorter overall survival. LncARSR promotes resistance to sunitinib by inhibiting AXL and MET expression through the adsorption of miR-34 and miR-449.

In an *in vivo* xenograft model of renal cell carcinoma, intravenous injection of l ASOs targeting lncARSR effectively restored sensitivity to Sunitinib treatment^132^. In patients with colon cancer, high expression of the lncRNA RAMS11 is associated with poor prognosis. Interestingly, an FDA-approved drug screen revealed that elevated RAMS11 expression confers resistance to fluorodeoxyuridine (FUDR) and topoisomerase inhibitors. Subsequent *in vitro* experiments demonstrated that CRISPR-mediated knockdown of RAMS11 expression increased cellular sensitivity to FUDR and topoisomerase inhibitors^133^, ^134^. Furthermore, lncRNAs also play a crucial role in the organ-specific target of tumor cells for metastasis. For instance, CRISPR-mediated knockdown of the lncRNA MAYA in breast cancer cells with bone metastasis significantly inhibited the activation of the YAP pathway, thereby impairing osteoclast differentiation and the bone resorption process^135^, ^136^. Additionally, research has revealed that m6A modifications on ncRNAs influence tumorigenesis and drug response, highlighting the therapeutic potential of targeting ncRNAs through m6A regulatory factors in cancer therapy^137^. The success of miRNA-based cancer research has spurred further investigations into other ncRNA families that may serve as potential therapeutic targets in cancer.

Despite the promising preclinical results, the translation of lncRNA-targeted therapies to the clinic faces several challenges. When targeting lncRNAs with ASO therapies, it is crucial to minimize off-target effects. Emerging evidence indicates that ASO can also induce premature transcription termination, potentially damaging target RNAs. This possibility should be carefully considered when evaluating the efficacy and safety of ASO therapies. The delivery of lncRNA-targeted therapeutics to tumor cells in the lung remains a significant hurdle due to the anatomical and physiological barriers. The abundance of lncRNAs exhibit overlapping sequences and functional similarities, complicating the design of targeted therapies that can selectively affect a single lncRNA without impacting others. While some lncRNAs have well-defined functions, many remain poorly understood. This lack of knowledge hinders the development of targeted therapies and complicates the prediction of potential off-target effects. Additionally, the potential off-target effects and toxicity of these therapies need to be thoroughly evaluated^138^. In conclusion, although the field of ncRNA-based therapeutics is still in its infancy, we are witnessing its burgeoning potential in cancer therapy. These therapeutics are anticipated to have a wide array of applications in the near future.

## Conclusions and future perspectives

Increasingly, studies have shown that lncRNAs play crucial roles in various essential cellular processes, and their roles in cancer, including NSCLC, are becoming increasingly apparent. LncRNAs interact with chromatin, RNA, and proteins, regulating gene expression at multiple stages and influencing important signaling path-ways. Numerous studies have demonstrated that lncRNAs participate in regulating processes such as proliferation, apoptosis, invasion, EMT, migration, and drug resistance in NSCLC cells. While certain lncRNAs have well-characterized mechanisms of action, many others remain to be fully elucidated. Concurrently, the application of multi-omics approaches, including encompassing genomics, transcriptomics, proteomics, and metabolomics, provides a comprehensive framework for investigating the functional roles of lncRNAs in NSCLC. This integrative perspective facilitates the identification of lncRNA interactions within cellular networks and their impact on critical signaling pathways, thereby enhancing our understanding of their contributions to tumor biology and supporting the development of more effective lncRNA-targeted therapies. Several lncRNAs show abnormal expression in NSCLC tissues and the circulation of NSCLC patients, suggesting their potential as diagnostic biomarkers for NSCLC. Unlike miRNAs, the expression levels of lncRNAs can better reflect the disease status, and their tissue-specific expression suggests potential diagnostic applications for specific diseases. LncRNAs are instrumental in modulating sensitivity to radiotherapy and chemotherapy in NSCLC, positioning them as promising targets for novel therapeutic agents that could revolutionize treatment strategies for NSCLC patients. To mitigate the risks associated with potential off-target effects of lncRNA-based therapies and to enhance therapeutic efficacy, advancements in drug delivery systems are critical. By optimizing delivery mechanisms, such as nanoparticle-based carriers and cell-affinity peptides, researchers seek to enhance the bioavailability and specificity of lncRNA-targeted drugs. This approach aims to address existing barriers that limit therapeutic potential, ultimately maximizing patient benefit and improving treatment outcomes.

In summary, lncRNAs play crucial roles in the development and progression of tumors, including NSCLC. Continued in-depth research into lncRNA could yield valuable insights and innovative approaches for the diagnosis and treatment of NSCLC. The development of novel diagnostic biomarkers and clinical therapeutic strategies targeting lncRNAs has the potential to guide future research directions and contribute to more effective cancer treatments.

## Figures and Tables

**Table 1:** Summarizing the roles and mechanisms of lncRNAs in NSCLC cell proliferation, cell cycle, and apoptosis

LncRNA	Expression	Mechanism	Function	References
PIK3CD-AS2	up	YBX1/p53	Proliferation, Apoptosis	42
MALAT1	up	miR-185-5p/MDM4	Proliferation, Apoptosis	43
MALAT1	up	MALAT1/FOXP3/GINS1	Proliferation	44
UPLA1	up	DSP/Wnt/β-catenin	Proliferation, Cycle	45
XIST	up	miR-744/RING1/Wnt/β-catenin	Proliferation	46
UFC1	up	UFC1/EZH2/PTEN/PI3K/Akt	Proliferation	47
LNC00525	up	miR-338-3p/IRS2	Proliferation	48
LNC00525	up	EZH2/RBMS2/p21	Proliferation, Cycle	49
AZIN1-AS1	up	miR4435-2HG/TGF-β1	Proliferation	50
AZIN1-AS1	up	miR-513b-5p/DUSP11	Proliferation	51
GAN1	down	miR-26a-5p/PTEN	Proliferation, Apoptosis	52
KTN1-AS1	up	KTN1-AS1/miR-130a-5p/PDPK1	Proliferation, Apoptosis	53
KTN1-AS1	up	miR-23b/DEPDC1	Proliferation	54
LNC00467	up	AKT	Proliferation	55
LNC00467	up	Wnt/β-catenin	Proliferation	56
LNC00467	up	miR-4779 and miR-7978	Proliferation, Apoptosis	57
RMRP	up	TGFBR1/SMAD2/SMAD3	Proliferation	58
LNC00301	up	EZH2/EAF2/pVHL/HIF-1α	Proliferation, Apoptosis	59

**Table 2:** Summarizing the roles and mechanisms of lncRNAs in NSCLC EMT, invasion and metastasis

LncRNA	Expression	Mechanism	Function	Reference
CRYBG3	up	Bub3 regulation	Migration	71
LNC01426	up	Hsa-miR-30b-3p/AZGP1 regulation	Migration, invasion	72
LNC01426	up	USP22/SHH regulation	Migration, EMT	73
LNC02678	up	EZH2/H3K27me3/CDKN1B regulation	Migration, invasion, EMT	74
LNC01234	up	HNRNPA2B1/miR-106b-5p/CRY2/c-Myc	Migration, invasion	75
LNC01234	up	EZH2/LSD1/BTG2 regulation	Migration	76
HOTAIR	up	miR-34a-5p/E-cadherin/vimentin/snail	Migration, invasion, EMT	77
LNC01123	up	miR-199a-5p/c-Myc pathway	Invasion, EMT	78

**Table 3:** Summarizing the roles and mechanisms of lncRNAs in NSCLC drug resistance

LncRNA	Expression	Mechanism	Function	Reference
SNHG15	up	MiR-451/MDR-1	Gefitinib resistance	111
CASC9	up	EZH2/DUSP1	Gefitinib resistance	100
CASC9	up	MiR-195-5p/FOXO3	Gefitinib resistance	112
UCA1	up	MiR-143/FOSL2	Gefitinib resistance	115
UCA1	up	MiR-495/NRF2 pathway	Cisplatin resistance	116
LNC01116	up	EZH2/CDKN1A	Cisplatin resistance	101
LNC01116	up	IFI44	Gefitinib resistance	43
FGD5-AS1	up	MiR-140-5p/WEE1 pathway	Cisplatin resistance	117
BLACAT1	up	STAT3	Afatinib resistance	114
BLACAT1	up	Cyclin D1	Cisplatin resistance	118
FOXD3-AS1	up	MiR-127-3p/MDM2 pathway	Cisplatin resistance	119
LNC00665	up	LNC00665/EZH2/CDKN1C	Cisplatin resistance	120
SPRY3	down	IGF2BP3	Radiosensitivity	121
KCNQ1OT1	up	MiR-372-3p/ATG5/ATG12	Radiosensitivity	104
KCNQ1OT1	up	MiR-27b-3p/HSP90AA1 pathway	Radiosensitivity	122
SNHG15	up	MiR-451/MDR-1	Gefitinib resistance	123
